# Effects of Deheading and Intestinal Removal on Protein Degradation and Quality Changes in Chilled *Litopenaeus vannamei*

**DOI:** 10.3390/foods15112034

**Published:** 2026-06-05

**Authors:** Shiliang Jia, Yating Zhao, Shuai Zhuang, Hong Zeng, Jie Chang, Shulai Liu, Yuting Ding, Xuxia Zhou, Yanbo Wang

**Affiliations:** 1 School of Food and Health, Beijing Technology and Business University, Beijing 100048, China; jiashiliang@zjut.edu.cn (S.J.); 20230903@btbu.edu.cn (S.Z.); zenghong@btbu.edu.cn (H.Z.); 2 Key Laboratory of Green, Low-Carbon and Efficient Development of Marine Fishery Resources, National R&D Branch Center for Pelagic Aquatic Products Processing (Hangzhou), College of Food Science and Technology, Zhejiang University of Technology, Hangzhou 310014, China; 221123260088@zjut.edu.cn (Y.Z.); slliu@zjut.edu.cn (S.L.); dingyt@zjut.edu.cn (Y.D.); 3 Jinghai Group Co., Ltd., Weihai 264307, China; jhjtcj@163.com; 4 Food Science Research Institute of Zhangzhou, Zhangzhou 363000, China

**Keywords:** *Litopenaeus vannamei*, endogenous enzymes, protein degradation, quality

## Abstract

This study evaluated the effects of deheading and intestinal removal on protein degradation and quality changes in chilled *Litopenaeus vannamei*. The results showed that shrimp subjected to heads removed (HR) and both heads and intestines removed (HIR) maintained better muscle integrity and sensory characteristics compared with intact (CK) and intestines removed (IR) shrimp. Furthermore, the HR and HIR treatments significantly inhibited the accumulation of total volatile basic nitrogen, trichloroacetic acid-soluble peptides, and bitter free amino acids (including His, Lys, Leu, Val, and Tyr), while maintaining consistently low activities of trypsin, cathepsin B, cathepsins B+L, and cathepsin D in the muscle throughout storage. Additionally, intrinsic fluorescence spectroscopy, SDS-PAGE, and microstructural analyses revealed that HR and HIR treatments significantly delayed muscle protein degradation and effectively preserved the structural integrity of muscle tissue. These findings suggest that quality deterioration of shrimp during chilled storage may be largely associated with the migration of endogenous proteases from the hepatopancreas in the cephalothorax into the muscle tissue.

## 1. Introduction

The Pacific white shrimp (*Litopenaeus vannamei*) is highly susceptible to rapid quality deterioration during post-harvest storage, resulting in substantial economic losses and limiting its commercial value. This deterioration is driven by a complex interplay of biochemical and microbial processes, including endogenous proteolysis, microbial proliferation, melanosis, and oxidative reactions [1,2]. As a high-protein, low-fat aquatic product, shrimp muscle is particularly vulnerable to structural destabilization, where the degradation of myofibrillar protein directly compromises texture, water-holding capacity, and overall sensory quality [3,4].

Emerging evidence suggests that quality deterioration in shrimp is initiated by endogenous enzymatic autolysis prior to microbial spoilage, highlighting the critical role of internal biochemical regulation in early post-mortem stages [5]. Zhou et al. [6] investigated the characteristics of endogenous enzymes in intact shrimps and shrimps with heads removed, as well as their effects on the muscle protein quality during freezing storage. Additionally, Wu et al. [7] studied the influence of shrimp intestines on shrimp muscles during refrigeration. They found that removing the shrimp heads/intestines can effectively delay the deterioration of shrimp muscle quality. The cephalothorax, especially the hepatopancreas, serves as a major reservoir of digestive enzymes, including trypsin-like proteases (TLPs), which are rapidly released upon tissue disruption after death. These enzymes migrate to the muscle and catalyze the hydrolysis of myofibrillar proteins, leading to fragmentation of myosin heavy chains and actin into peptides and free amino acids [8]. Concurrently, lysosomal enzymes such as cathepsins (B, D, and L) further exacerbate intracellular protein degradation following membrane destabilization [9]. This enzymatic cascade disrupts the hierarchical structure of muscle, affecting both molecular conformation and sarcomere integrity, ultimately leading to texture softening and structural collapse. In parallel, microbial proliferation constitutes a secondary but amplifying driver of quality deterioration. Following death, the cephalothorax and intestinal tract transition from protective barriers to nutrient-rich niches that facilitate rapid microbial growth [10]. The intestinal microbiota, in particular, plays a pivotal role in protein catabolism by secreting extracellular proteases and interacting with degradation metabolites [11]. Spoilage-associated genera such as *Vibrio* and *Aliivibrio* have been shown to accelerate protein degradation and off-flavor formation during storage [12]. Moreover, endogenous proteolysis generates peptides and amino acids that serve as substrates for microbial metabolism, establishing a positive feedback loop between enzymatic degradation and microbial activity. This synergy accelerates physicochemical deterioration, including increases in total volatile basic nitrogen (TVB-N), accumulation of TCA-soluble peptides, and loss of structural integrity [5].

Despite these advances, a critical gap remains: the relative contributions and mechanistic interplay of different anatomical sources of endogenous enzymes—particularly the cephalothorax and intestine—are not well resolved. In industrial practice, deheading and intestinal removal are widely applied to improve shrimp quality and shelf life. However, the extent to which these processing steps modulate endogenous enzyme activity and subsequent protein degradation pathways has not been systematically elucidated. The cephalothorax, especially the hepatopancreas, contains abundant digestive enzymes such as trypsin-like proteases that are released post-mortem and accelerate myofibrillar protein hydrolysis. The intestine may serve as an additional enzymatic source. Together with intrinsic muscle proteases (e.g., cathepsins), these form a multi-source enzymatic system driving structural degradation and texture softening. Removal of these tissues may therefore attenuate proteolysis and delay quality loss. To test this, shrimp subjected to deheading (HR), intestinal removal (IR), and combined treatment (HIR) were compared with intact samples (CK). Protease activities (trypsin and cathepsins), along with biochemical, structural, and physicochemical quality attributes, were systematically analyzed to elucidate the mechanistic link between anatomical processing and protein degradation.

## 2. Materials and Methods

### 2.1. Chemical Reagents

Sodium chloride, sodium hydroxide, boric acid, magnesium oxide, hydrochloric acid, trichloroacetic acid, anhydrous ethanol, dimethyl sulfoxide (DMSO), Bis (2-hydroxyethyl) amino (tris (hydroxymethyl) methane) (Bis-Tris), calcium chloride (CaCl2), acetic acid were purchased from Sinopharm Chemical Reagent Co., Ltd. (Shanghai, China). Sodium dodecyl sulfate and 7-Amino-4-methylcoumarin (AMC), ethylene diamine tetraacetic acid (EDTA), dithiothreitol (DTT), tris (hydroxymethyl) aminomethane and hemoglobin were obtained from Hangzhou Jigong Biotechnology Co., Ltd (Hangzhou, China). N-Benzoyl-DL-arginine-4-nitroanilidehydrochloride (BAPNA) was obtained from Hangzhou Qichuang Scientific Equipment Co., Ltd. (Hangzhou, China). Z-Arg-Arg-AMC and Z-Phe-Arg-AMC were obtained from Nanjing YuanPeptide Biotechnology Co., Ltd. (Nanjing, China). Glutaraldehyde solution was supplied by Wuhan Servicebio Technology Co., Ltd. (Wuhan, China). Osmium tetroxide and uranyl acetate were purchased from Ted Pella Inc. (Redding, CA, USA) and Structure Probe Inc. (West Chester, PA, USA), respectively. Lead nitrate was obtained from Sigma-Aldrich (St. Louis, MO, USA). All reagents were of analytical or HPLC grade. 

### 2.2. Sample Preparation

Fresh *Litopenaeus vannamei* were purchased from a local aquatic market in Huzhou, China. The shrimp were of uniform size, with an average weight of approximately 20 g. After being rinsed twice with clean water, the samples were randomly divided into four groups: CK (intact shrimp without treatment), HR (head-removed), IR (intestine-removed), and HIR (both head- and intestine-removed). The shells were retained in all groups. Samples were then placed in sterile disposable trays, sealed in sterile sampling bags, and all the samples were stored in a 4 °C refrigerator for 6 days of refrigeration. Quality parameters were determined every 24 h during chilled storage.

### 2.3. Sensory Analysis

The sensory characteristics were evaluated by six students majoring in food science. The evaluation criteria included appearance, texture, odor, and overall acceptability. The sensory characteristics with description and scores, ranging from 0 to 9, are shown in Table 1. These six students received prior training in sensory evaluation for shrimp quality to ensure the repeatability of the scores.

### 2.4. Determination of Texture

For each group, five shrimp were randomly selected for testing. The muscle tissue from the first and second abdominal segments of the shrimp was excised, cut into 1 cm^3^ cubes, and then analyzed using a texture profile analysis (TPA) unit (TA.XT PlusC, Stable Micro Systems, London, UK) equipped with a flat-ended cylindrical probe of 36 mm diameter. Instrument parameters: 3.00 mm/s of pre-test speed, 1.00 mm/s of test speed, 1.00 mm/s of return speed, 5 mm of compression distance, 5 s of hold time, and 5 g of trigger force. TPA results were expressed in terms of hardness, elasticity, and chewiness.

### 2.5. Determination of Color

From each group, five shrimp were randomly selected. The second abdominal segment of each specimen was used for color index evaluation, measuring lightness (L*), redness (a*), and yellowness (b*). Ten replicate measurements were performed on the designated area using a colorimeter (NR10QC, 3nh, Guangzhou, China), and the average value was calculated to represent the color characteristics.

### 2.6. Determination of the Activity of Trypsin

Trypsin activity was measured using BAPNA as substrates according to the methods of Peng et al. [8] with slight modifications. Briefly, 2 g of hepatopancreas or minced shrimp muscle was homogenized with 6 mL of pre-cooled (4 °C) separation buffer (0.01 mol/L Tris-HCl, pH 8.0) at 7000 rpm for 20 s. The homogenate was centrifuged at 10,000 rpm for 20 min at 4 °C, and the resulting supernatant was collected as the crude trypsin extract. A mixture containing 200 μL of the extract, 200 μL of distilled water, 1 mL of 50 mmol/L Tris-HCl (pH 8.0) containing 10 mmol/L CaCl_2_, and 200 μL of BAPNA (2 mg/mL) was blended thoroughly and incubated at 25 °C for 15 min. The reaction was terminated by adding 200 μL of 30% (*v*/*v*) acetic acid solution. The blank was treated identically, except that BAPNA was added after the addition of acetic acid. The mixture was centrifuged at 12,000 rpm for 5 min, and the supernatant was collected to measure the absorbance at 410 nm (A_410_). The trypsin-like protease activity unit (U) is defined as the amount of enzyme required to release 1 μmol of ρ-nitroaniline per minute (1 μmol/min).

### 2.7. Determination of the Activities of Cathepsin B, B+L and D

The crude enzyme solution and the determination of the activities of cathepsin B and B+L were extracted and slightly modified by referring to the method of Xiong et al. [13]. Briefly, 2 g of minced shrimp muscle samples were added to 8 mL of pre-cooled (4 °C) 20 mmol/L Tris-HCl (pH 7.5) buffer, homogenized at 10,000 rpm for 1 min, and then centrifuged at 10,000 rpm at 4 °C for 20 min. The supernatant was the crude enzyme solution, which should be aliquoted and stored at −80 °C. The fluorescent synthetic substrates used for cathepsin B and B+L activities were Z-Arg-Arg-AMC and Z-Phe-Arg-AMC, respectively. Substrate stock solutions (3 mmol/L) were prepared using DMSO and stored at −20 °C for later use. Before use, the substrates were diluted to 90 μmol/L with deionim pH 7.0). The mixture was immediately placed in an ice bath, and the fluorescence intensity was measured within 10 min. For the blank group, the crude enzyme extract was replaced with buffer solution. The excitation and emission wavelengths for fluorescence measurement were 380 nm and 460 nm, respectively. The enzyme activity unit (U) is defined as the amount of enzyme required to hydrolyze the substrate and release 1 nmol of AMC product per minute at 30 °C (1 nmol/min).

The determination of cathepsin D activity referred to the method of Chen et al. [14] with slight modifications. A mixture of 100 μL of 0.2 mol/L citrate buffer (pH 3.0) and 100 μL of 25 mg/mL bovine hemoglobin was pre-warmed at 30 °C for 10 min. Then, 200 μL of crude enzyme extract was added, and the mixture was incubated at 37 °C for 1 h. The reaction was stopped by adding 800 μL of 10% (*w*/*v*) TCA solution. After centrifugation at 10,000 rpm for 5 min, the supernatant was collected, and the peptide content in the supernatant was determined using the Lowry method. The enzyme unit (U) is defined as the amount of enzyme required to release 1 nmol of tyrosine per minute at 37 °C (1 nmoL/min).

### 2.8. Determination of Total Volatile Basic Nitrogen (TVB-N)

TVB-N was determined according to Wang et al. [15] as follows: 5 g of shrimp muscle was mixed with 45 mL of distilled water, homogenized at 7000 r/min for 20 s, left to stand at 4 °C for 30 min, and then centrifuged at 8000× *g* for 10 min. The supernatant was combined with 0.5 g of MgO and distilled for 5 min. The distillate was absorbed in boric acid solution and titrated with 0.01 mol/L HCl. Three parallel measurements were performed per group. The TVB-N value (mg N/100 g) was calculated from the HCl consumption.

### 2.9. Determination of Trichloroacetic Acid (TCA)-Soluble Peptides

TCA-soluble peptides were determined as described by Zeng et al. [16] with slight modifications. Briefly, 2.0 g of shrimp muscle was homogenized with 18 mL of 5% (*w*/*v*) trichloroacetic acid at 7000 rpm for 30 s. The homogenate was incubated on ice for 30 min and then centrifuged at 8000 rpm for 10 min at 4 °C. The supernatant was collected, and the peptide concentration was measured using the Lowry method. Results are expressed as μmol tyrosine/g shrimp muscle.

### 2.10. Extraction of Protein

Myofibrillar proteins were extracted according to the method of Yuan et al. [17] with slight modifications. Briefly, 2.0 g of shrimp muscle was mixed with 20 mL of solution A and homogenized. The mixture was centrifuged at 9000 rpm for 10 min (4 °C). The supernatant was discarded, and the pellet was resuspended in 20 mL of solution B and homogenized again. The suspension was incubated at 4 °C for 1 h and then centrifuged under the same conditions. The supernatant was collected as the myofibrillar protein extract. Solution A consisted of 20 mL of 0.05 M NaCl-20 mM Tris-maleate buffer (pH 7.0), and solution B was 0.6 M NaCl-20 mM Tris-maleate buffer (pH 7.0).

### 2.11. Intrinsic Fluorescence

Intrinsic fluorescence was extracted following the protocol described by Yu et al. [18], with slight modifications. The concentration of the extracted myofibrillar protein solution was adjusted to 0.5 mg/mL, and the myofibrin samples were scanned at high speed using a fluorescence spectrometer (Flugromax-plus-p, Horiba (China) Trading Co., Ltd., Shanghai, China). The specific conditions were as follows: the excitation wavelength was 295 nm, both the excitation slit and the emission slit were 1 nm, the scanning range was 300 to 400 nm, the scanning speed was 0.25 s per time.

### 2.12. Determination of Protein Degradation

The extracted myofibrillar protein solution was diluted to 5 mg/mL and subjected to SDS-PAGE according to the method of Jia et al. [19] with slight modifications. The protein sample was mixed with loading buffer at a 4:1 ratio, vortexed, and then heated in a boiling water bath for 2 min. A 12% separating gel and a 4% stacking gel were used, and 10 μL of the prepared sample was loaded per well. Electrophoresis was performed at 80 V until the dye front migrated approximately 1 cm into the separating gel. The gel was stained with Coomassie Brilliant Blue solution for 20 min and then destained until the protein bands became clearly visible.

### 2.13. Determination of Microstructure of Muscle Fibers

Observation of myofibril microstructure was performed according to the protocol described by Zhao et al. [20]. Briefly, dorsal muscle tissue from the second abdominal segment of shrimp was cut into 1.0 mm^3^ pieces and fixed with 2.5% glutaraldehyde. The specimens were then post-fixed with 1% osmium tetroxide for 2 h, rinsed three times with 0.1 M phosphate buffer, and dehydrated through a graded ethanol series (20 min each step). This was followed by two additional dehydrations with 100% acetone (15 min each). The dehydrated samples were subsequently infiltrated, embedded, polymerized, oriented, and sectioned into ultrathin sections. After staining, the ultrathin sections were examined using a transmission electron microscope (TEM; HT7800, Hitachi, Tokyo, Japan).

### 2.14. Determination of Free Amino Acid Content

Free amino acids (FAAs) were determined following the method described by Lin et al. [21], with slight modifications. Briefly, 2 g of intact shrimp muscle was homogenized with 15 mL of cold (4 °C) 15% TCA solution and the mixture was allowed to stand for 2 h. The mixture was then centrifuged at 10,000 rpm for 15 min at 4 °C. 10 mL of the supernatant was taken, and the pH was adjusted to 2.0 with 10 mol/L NaOH and 1 mol/L NaOH. The volume was made up to 20 mL with pure water. The solution was filtered through a 0.22 μm microporous membrane, aliquoted, and stored in a −80 °C refrigerator for subsequent test analysis. The composition and content of free amino acids in the sample solution were measured by an automatic amino acid analyzer (LA8080, Hitachi, Japan).

### 2.15. Statistical Analysis

In color and texture analyses, each individual shrimp was treated as one biological replicate, and five repeated measurements were performed per shrimp. For all other analyses, three shrimp were pooled as one replicate, resulting in three independent replicates for each treatment group at each storage time point (*n* = 3). All measurements were performed on the same individuals. Data processing was performed using Microsoft Excel 2021, and graph plotting was carried out using Origin 2021. All experimental data were analyzed by one-way analysis of variance (ANOVA), followed by Duncan’s multiple range test, with the significance threshold set at *p* < 0.05. Prior to ANOVA, the Shapiro–Wilk test and Levene’s test were used to assess the assumptions of normality and homogeneity of variance, respectively. The same statistical procedure was applied to all measured variables. All analyses were performed using SPSS version 26.0, and the results are expressed as mean ± standard deviation.

## 3. Results

### 3.1. Sensory Analysis

**Figure 1:**
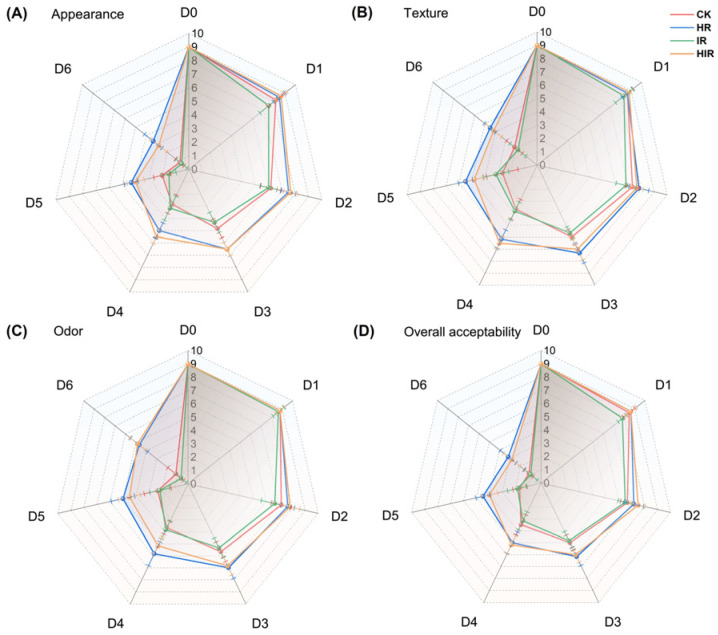
Changes in sensory indicators (Appearance (**A**), Texture (**B**), Odor (**C**) and Overall acceptability (**D**)) of *Litopenaeus vannamei* under different treatments during storage at 4 °C. (Note: CK, HR, IR, and HIR represent the pretreatment of shrimp as intact shrimp, shrimp with head removed, shrimp with intestine removed, and shrimp with head and intestine removed, respectively, followed by storage at 4 °C). D3-CK, D3-HR, D3-IR, and D3-HIR: CK, HR, IR, and HIR samples on day 3; D6-CK, D6-HR, D6-IR, and D6-HIR: CK, HR, IR, and HIR samples on day 6.

Sensory attributes are critical indicators of consumer acceptance and quality deterioration in fresh shrimp [22]. Figure 1 shows the sensory changes in *Litopenaeus vannamei* under different treatments during storage at 4 °C. Appearance (A), texture (B), odor (C), and overall acceptability (D) were evaluated, with scores below 4 considered unacceptable. All samples showed excellent initial quality, followed by progressive sensory decline during storage. By day 4, the CK and IR groups had dropped below the acceptability threshold in all attributes, whereas the HR and HIR groups maintained acceptable appearance and overall acceptability until day 6, with odor and texture scores remaining above 4 throughout storage. Overall, the deheaded groups (HR and HIR) exhibited superior sensory stability during chilled storage. These results indicated that the improved sensory quality of deheaded shrimp was mainly attributed to the removal of the cephalothorax. The cephalothorax serves as major source of polyphenol oxidase (melanosis) [2], endogenous proteases (texture softening) [14], off-odor precursors (ammonia-/iodine-like) [10], and approximately 50–80% of spoilage-related microorganisms [9]. Consequently, the retained head in the CK and IR groups might be promote enzymatic browning, microbial growth, protein degradation, and lipid oxidation, thereby accelerating sensory deterioration during storage. Although deheading significantly delayed quality loss, residual enzymes and microorganisms in muscle tissue still gradually induced spoilage during prolonged chilled storage.

### 3.2. Texture Analysis

**Figure 2:**
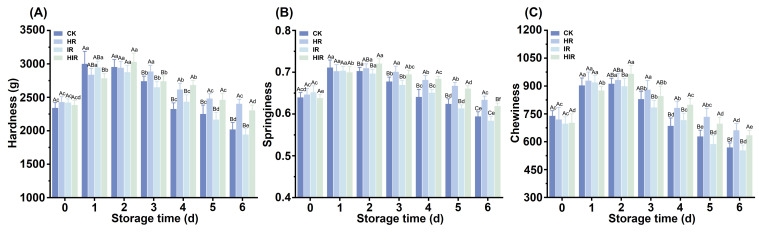
Changes in texture of Hardness (**A**), Springiness (**B**) and Chewiness (**C**) of *Litopenaeus vannamei* under different treatments during storage at 4 °C. (Note: Different capital letters indicate significant differences between groups (*p* < 0.05); while different lowercase letters indicate significant differences within groups (*p* < 0.05); CK, HR, IR, and HIR represent the pretreatment of shrimp as intact shrimp, shrimp with head removed, shrimp with intestine removed, and shrimp with head and intestine removed, respectively, followed by storage at 4 °C).

Figure 2 illustrates the changes in the textural properties of *Litopenaeus vannamei* subjected to different treatments during storage at 4 °C. The hardness, springiness, and chewiness of all groups initially increased and then significantly decreased during storage (*p* < 0.05). The initial increase in these parameters may be attributed to the post-mortem contraction of myofibrils, which leads to muscle tightening and rigidity during the early chilling phase [23]. The subsequent decline is primarily associated with the proteolytic activity of endogenous and microbial proteases, including intestinal proteases, cathepsins, trypsin, and collagenases [24,25,26]. Notably, starting from day 3 of storage, the hardness, springiness, and chewiness of the CK and IR groups began to decrease significantly. In contrast, the overall textural properties of the HR and HIR groups were superior to those of the CK and IR groups. Li et al. [27] also found that the shear force and hardness of the intact shrimp samples were significantly lower than those of the beheaded shrimp samples. However, the textural differences between the CK and IR groups, as well as between the HR and HIR groups, were relatively limited. These findings indicate that deheading plays a dominant role in texture preservation, which may prevent the diffusion of endogenous proteases from the cephalothorax into muscle tissue, thereby delaying proteolysis and alleviating the deterioration of hardness, springiness, and chewiness during chilled storage.

### 3.3. Color Analysis

Color is an important and intuitive indicator of seafood freshness and quality. Table 2 shows the color changes in *Litopenaeus vannamei* under different treatments during storage at 4 °C. During the early storage stage, the L* value (lightness) increased significantly in all groups, mainly due to post-mortem cell disruption and reduced water-holding capacity, which caused surface water exudation and enhanced light reflection [28]. Structural collapse of muscle fibers also increased surface opacity, further improving perceived brightness. As storage progressed, L* values gradually decreased. Notably, the HR and HIR groups consistently maintained significantly higher L* values than the CK and IR groups. This may be mainly attributed to the removal of the cephalothorax, where polyphenol oxidase (PPO) is highly concentrated, thereby effectively reducing enzymatic browning. The a* (redness) and b* (yellowness) values gradually increased in all groups during storage, indicating progressive red and yellow discoloration. This phenomenon might be associated with astaxanthin release from disrupted pigment-protein complexes [29], as well as lipid oxidation [30,31] in the cephalothorax. Therefore, deheading may effectively remove major sources of PPO, endogenous enzymes, and lipid oxidation substrates, significantly delaying color deterioration. However, residual microbial activity and oxidative reactions still gradually induced discoloration during prolonged storage. Overall, the HR and HIR groups exhibited markedly superior color stability compared with the CK and IR groups throughout chilled storage.

### 3.4. Trypsin Activity Analysis

**Figure 3:**
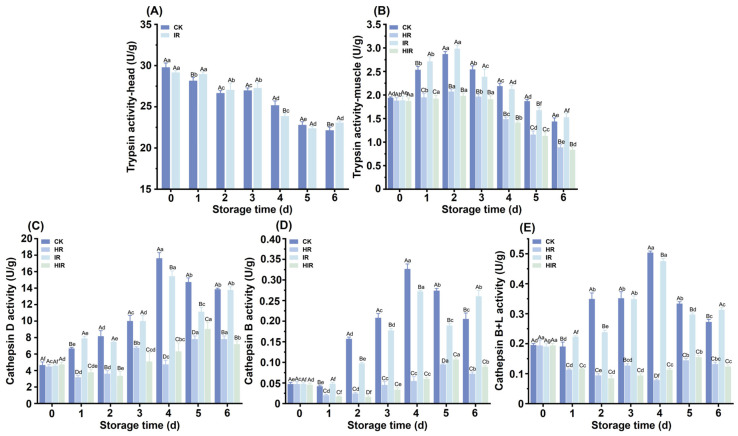
Changes in trypsin activity in the head (**A**) and muscle (**B**), cathepsin D activity (**C**), cathepsin B activity (**D**) and cathepsin B+L activity (**E**) of *Litopenaeus vannamei* under different treatments during storage at 4 °C. (Note: Different capital letters indicate significant differences between groups (*p* < 0.05); while different lowercase letters indicate significant differences within groups (*p* < 0.05); CK, HR, IR, and HIR represent the pretreatment of shrimp as intact shrimp, shrimp with head removed, shrimp with intestine removed, and shrimp with head and intestine removed, respectively, followed by storage at 4 °C).

Trypsin is a major serine protease in shrimp, with the cephalothorax serving as its primary reservoir [32]. As shown in Figure 3A, trypsin activity in the cephalothorax of the CK and IR groups gradually decreased during chilled storage. This decline may be mainly associated with oxidative stress, microbial proliferation, endogenous proteolysis, and postmortem acidification, which collectively accelerate trypsin denaturation and degradation during storage [2,33,34]. Furthermore, the hepatopancreas is unable to synthesize new trypsin after death to compensate for enzyme depletion [8]. Zhou et al. [6] also demonstrated that the trypsin activity in the cephalic gland of the sword shrimp showed a gradually decreasing trend during storage. As shown in Figure 3B, muscle trypsin activity in the HR and HIR groups remained consistently lower than that in the CK and IR groups throughout storage. In contrast, the CK and IR groups exhibited an initial increase followed by a gradual decline, consistent with the findings of Sriket et al. [35]. This pattern suggests that structural disintegration of the cephalothorax released trypsin-rich contents, allowing trypsin to migrate into adjacent muscle tissue and temporarily elevate enzyme activity [34]. Subsequently, trypsin activity declined due to depletion of the cephalothorax enzyme reservoir and progressive enzyme inactivation under acidic, oxidative, and microbial conditions in the muscle [36].

### 3.5. The Activities of Cathepsin B, B+L and D Analysis

Cathepsin D is an aspartic protease that preferentially hydrolyzes peptide bonds formed by hydrophobic amino acids under acidic conditions [37]. Cathepsins B and L are cysteine proteases capable of degrading key structural proteins such as myosin, actin, tropomyosin, and collagen [38,39]. Figure 3C–E presents the changes in cathepsin D, B, and B+L activities in *Litopenaeus vannamei* during storage at 4 °C. In the CK and IR groups, all three enzymes exhibited a trend of initial increase followed by gradual decline, consistent with the findings of Zhu et al. [40]. In contrast, the HR and the HIR groups showed a trend of initial decrease, subsequent increase, and final decline. The initial decrease may be attributed to the inhibitory effect of low-temperature storage on enzyme activity [41]. The activities of cathepsins D, B, and B+L in the CK and IR groups reached peak values on day 4, whereas those in the HR and HIR groups peaked later on day 5. Throughout storage, cathepsin activities in the HR and HIR groups remained significantly lower than those in the CK and IR groups. The early increase in cathepsin activity was mainly associated with post-mortem energy depletion and lysosomal membrane disruption, which may be promote the release of cathepsins into muscle tissue [42]. Simultaneously, post-mortem glycolysis reduced muscle pH to a mildly acidic range favorable for cathepsin activation [43,44,45]. As storage progressed, enzyme activities gradually declined due to protein denaturation during prolonged cold exposure and pH rebound caused by microbial metabolism and accumulation of alkaline compounds such as ammonia and amines [34,46]. The significantly higher cathepsin activities in the CK and IR groups were primarily related to the retained cephalothorax. The hepatopancreas is rich in endogenous digestive enzymes, including proteases and lipases, at levels much higher than those in muscle tissue. During storage, structural deterioration of the cephalothorax likely facilitated enzyme leakage and diffusion into adjacent muscle, thereby intensifying proteolysis [30,31]. In addition, stronger microbial and enzymatic activities in intact shrimp accelerated pH changes and muscle deterioration. Therefore, deheading effectively reduced the overall proteolytic burden and delayed the peak activation of cathepsins, contributing to improved muscle stability during chilled storage.

### 3.6. TCA-Solution Peptides Analysis

**Figure 4:**
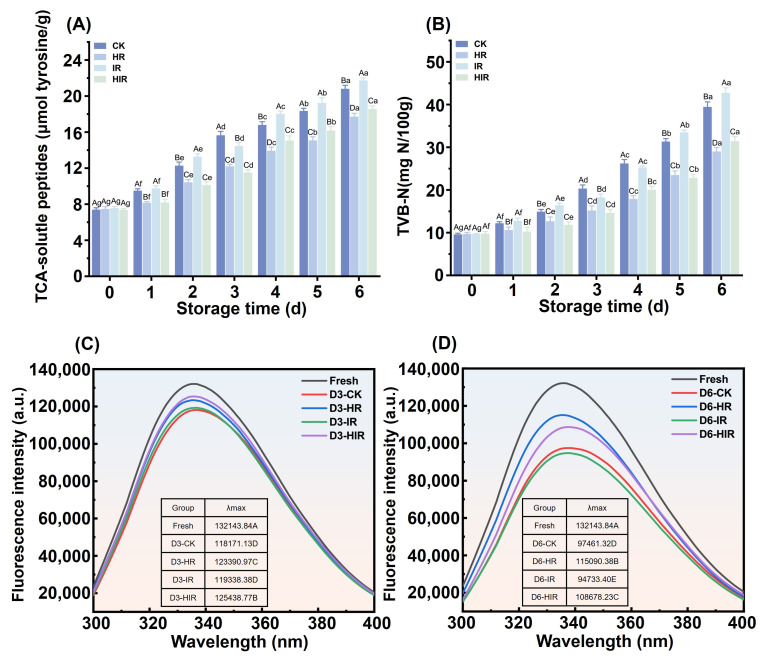
Changes in TCA-soluble peptides (**A**), TVB-N (**B**) and fluorescence intensity (**C**,**D**) of *Litopenaeus vannamei* under different treatments during storage at 4 °C. (Note: Different capital letters indicate significant differences between groups (*p* < 0.05); while different lowercase letters indicate significant differences within groups (*p* < 0.05); Fresh: fresh shrimp muscle samples; D3-CK, D3-HR, D3-IR, and D3-HIR: CK, HR, IR, and HIR samples on day 3; D6-CK, D6-HR, D6-IR, and D6-HIR: CK, HR, IR, and HIR samples on day 6; CK, HR, IR, and HIR represent the pretreatment of shrimp as intact shrimp, shrimp with head removed, shrimp with intestine removed, and shrimp with head and intestine removed, respectively, followed by storage at 4 °C).

Trichloroacetic acid (TCA)-soluble peptides is an important indicator of proteolytic degradation, with higher levels reflecting more extensive protein breakdown. As shown in Figure 4A, the TCA-soluble peptide content gradually increased in all groups during chilled storage, mainly due to the combined action of endogenous and microbial proteases [47]. At the end of storage, the contents reached 20.91 ± 0.21 (CK), 18.02 ± 0.35 (HR), 21.69 ± 0.28 (IR), and 18.99 ± 0.42 μmol tyrosine/g (HIR). Notably, the HR and HIR groups exhibited significantly lower peptide accumulation than the CK and IR groups, indicating that deheading effectively alleviated proteolytic deterioration. Kim et al. [48] reported that intact shrimp were more susceptible to spoilage and freshness loss during storage. The cephalothorax is rich in proteins, lipids, and highly active endogenous proteases, providing favorable conditions for microbial growth and continuous enzymatic hydrolysis [10]. During storage, these proteases may diffuse into adjacent muscle tissue and accelerate the degradation of myofibrillar proteins such as myosin and actin into small peptides and free amino acids [14,19,49]. In contrast, deheaded shrimp (HR and HIR) contained substantially lower residual enzymatic activity, resulting in slower proteolysis and reduced accumulation of TCA-soluble peptides throughout storage.

### 3.7. Total Volatile Base Nitrogen (TVB-N) Analysis

TVB-N is an important freshness indicator that reflects the degradation of proteins and non-protein nitrogenous compounds into volatile alkaline substances, such as ammonia, dimethylamine, and trimethylamine, through microbial and enzymatic activities [9,50]. According to the Chinese standard GB/T 2733-2015 [51], the acceptable limit for marine shrimp is ≤30 mg N/100 g. As shown in Figure 4B, TVB-N values in all groups gradually increased during chilled storage, showing a trend similar to that of TCA-soluble peptides. By day 5, the TVB-N values of the CK (30.35 ± 0.43 mg N/100 g) and IR (33.15 ± 0.86 mg N/100 g) groups exceeded the acceptable limit, whereas the HR (23.63 ± 0.70 mg N/100 g) and HIR (22.69 ± 0.70 mg N/100 g) groups remained significantly lower. The higher TVB-N accumulation in the CK and IR groups was mainly attributed to the retained shrimp head, which serves as a major site for microbial growth and amino acid decomposition [10]. The high initial microbial load in the cephalothorax, combined with the release of free amino acids from enzymatic hydrolysis in the head, allows psychrotrophic spoilage bacteria (e.g., *Pseudomonas*, *Shewanella*) to proliferate, migrate to the muscle via exudate, and generate TVB-N [10,34,52]. In contrast, deheading effectively removed the major contamination source, reduced the initial microbial burden, and slowed amino acid accumulation in muscle tissue, thereby significantly suppressing microbial proliferation and TVB-N formation during storage. Consequently, deheaded shrimp remained within the acceptable TVB-N limit for approximately 2 days longer than head-retained shrimp, indicating that deheading effectively delayed spoilage and extended chilled shelf life.

### 3.8. Protein Tertiary Structure Analysis

The tertiary structure of myofibrillar proteins is commonly assessed by measuring the intrinsic fluorescence intensity (FI) of tryptophan residues [53]. Tryptophan is the main contributor to intrinsic protein fluorescence. When a protein unfolds, tryptophan residues become exposed to the hydrophilic environment, typically decreasing fluorescence intensity and thus reflecting protein denaturation [54]. As shown in Figure 4C,D, the FI of myofibrillar proteins in all groups decreased during storage. This decline suggests protein aggregation, which may cause some exposed hydrophobic residues to refold into the protein core, leading to fluorescence quenching [5,55]. Notably, the CK and IR groups showed a significantly steeper decline and maintained lower overall FI than the HR and HIR groups throughout storage. This directly demonstrates that proteins in head-retaining groups (CK, IR) underwent more rapid and severe denaturation and degradation. This may be attributed to the postmortem release and gradual penetration of endogenous enzymes from the shrimp head into the muscle, where hydrolysis disrupts the compact tertiary structure, exposing previously buried tryptophan residues to the hydrophilic environment [5,41]. In contrast, the head-removed groups (HR, HIR) experienced less protein degradation, a more stable tryptophan microenvironment, and less quenching, thus retaining higher FI. These findings are consistent with the changes in TCA-soluble peptide content and TVB-N levels reported above.

### 3.9. SDS-PAGE Analysis

**Figure 5:**
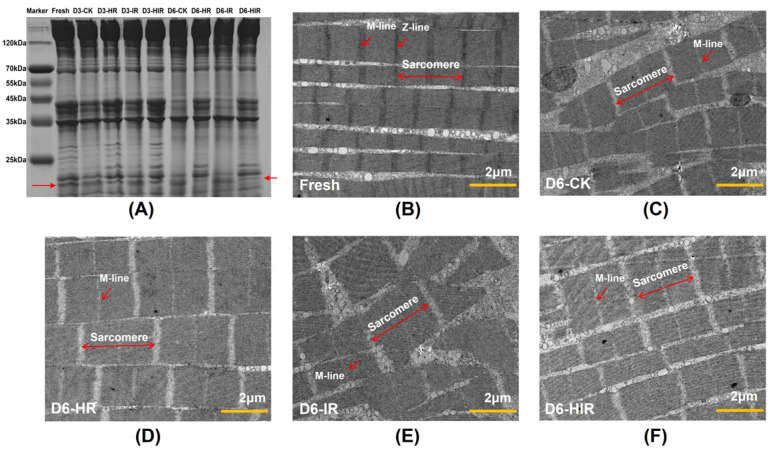
Myofibrillar protein degradation analysis based on SDS-PAGE (**A**) and myofibril microstructure (**B**–**F**) of *Litopenaeus vannamei* under different treatments during storage at 4 °C. (Note: Marker: molecular weight marker, Fresh: fresh shrimp muscle samples; D3-CK, D3-HR, D3-IR, and D3-HIR: CK, HR, IR, and HIR samples on day 3; D6-CK, D6-HR, D6-IR, and D6-HIR: CK, HR, IR, and HIR samples on day 6; (**B**): myofibril from fresh shrimp on day 0; (**C**–**F**): myofibril from CK, HR, IR, and HIR samples on day 6; CK, HR, IR, and HIR represent the pretreatment of shrimp as intact shrimp, shrimp with head removed, shrimp with intestine removed, and shrimp with head and intestine removed, respectively, followed by storage at 4 °C).

Sodium dodecyl sulfate-polyacrylamide gel electrophoresis (SDS-PAGE) is widely used to assess protein molecular weight and structural integrity. Key myofibrillar proteins include myosin heavy chain (MHC, ~220 kDa), actin (AC, ~43 kDa), troponin (~35 kDa), and myosin light chain (MLC, 16–25 kDa) [56,57]. As shown in Figure 5A, compared to the Fresh group, the actin and MLC bands in all four treatment groups became noticeably fainter after 3 and 6 days of storage, indicating ongoing proteolytic degradation likely due to endogenous and microbial proteases. This aligns with H. Li et al. [58], who observed similar weakening of MHC, actin, troponin-T, and MLC bands in mirror carp during extended storage. Concurrently, a distinct band near 18 kDa (red arrow in Figure 5A) intensified over time, suggesting accumulation of low-molecular-weight peptides from larger myofibrillar protein hydrolysis. Notably, the degree of fading varied among groups. After 6 days, the HR and HIR groups retained markedly stronger bands in the 38–43 kDa and 20–28 kDa regions than the CK and IR groups, while the ~18 kDa band was more intense in the CK and IR groups. This indicates that during storage, endogenous proteases from the shrimp head likely migrated into the muscle, driving more severe protein breakdown into small peptides or free amino acids. Similar conclusions were reached by Zhou et al. [6] and Li et al. [27]. The SDS-PAGE results directly support the earlier findings that the presence of the shrimp head leads to higher enzymatic activity, lower fluorescence intensity, and more severe texture deterioration.

### 3.10. Microstructure Observations

Microstructural analysis revealed distinct differences in the maintenance of muscle tissue structural stability among the treatment groups during cold storage (Figure 5B–F). Fresh shrimp muscle exhibited a tightly ordered lattice of myofibrils with distinct sarcomeres, intact Z-lines, and visible M-lines (Figure 5B). After 6 days of storage, severe myofibrillar fragmentation and expanded extracellular spaces were observed in the CK and IR groups, where the myofibrillar structure became highly irregular. In contrast, the HR and HIR treatments largely preserved the structural order of the myofibrils, with significantly less fragmentation. This preservation is likely attributable to the removal of the cephalothorax, which prevented the migration of endogenous proteases from the head region into the muscle, thereby mitigating damage to the myofibrils. Li et al. [27] also found that the structure of the deheaded shrimp tissue showed a relatively better organization compared to that of the intact shrimp samples. Xu et al. [59] attributed the deterioration of microstructure observed during cold storage to the cleavage of connective tissue between collagen and muscle fibers by cathepsins. The consistency among microstructural, electrophoretic (SDS-PAGE), and physicochemical data underscores the profound impact of endogenous enzymes from the shrimp head on muscle quality deterioration.

### 3.11. Free Amino Acid Content Analysis

The profile and concentration of free amino acids (FAAs) serve as effective indicators of the extent of protein degradation, oxidative status, and involvement in biochemical reactions [60]. They significantly influence the flavor profile of aquatic products, contributing to sourness, bitterness, sweetness, and umami [61]. FAAs affect volatile compound formation primarily through pathways such as oxidation, transamination, deamination, decarboxylation, and Strecker degradation [62]. Certain amino acids with active groups, particularly in their free form, are susceptible to oxidation. This not only depletes characteristic amino acids but also allows oxidation products to further react, influencing the overall flavor profile [63]. For instance, sulfur-containing amino acids like cysteine and methionine, which contain reactive -SH groups, are readily oxidized [64]. As shown in Table 3, the concentrations of histidine (His), lysine (Lys), phenylalanine (Phe), tyrosine (Tyr), methionine (Met), isoleucine (Ile), leucine (Leu), and valine (Val) increased gradually over the storage period. Notably, His, Lys, Val, Leu, and Tyr are typically associated with bitter taste, and their levels in the headed (HR) and headed-and-intestine-removed (HIR) groups were significantly lower than those in the control (CK) and intestine-removed (IR) groups. These results indicate that endogenous proteases and microbial activities may gradually break down the myofibrillar proteins after the shrimp’s death. In the CK and IR groups, the concentrated endogenous proteases from the cephalothorax may be continuously diffuse into the muscle, resulting in a markedly faster proteolysis rate and more severe protein degradation. Consequently, the accumulation rates of these eight amino acids were substantially higher in the CK and IR groups compared to the HR and HIR groups.

## 4. Discussion

The present results demonstrated that the cephalothorax played a critical role in the quality deterioration of *Litopenaeus vannamei* during chilled storage. Compared with the CK and IR groups, the HR and HIR groups consistently maintained higher sensory scores, better texture properties, and superior color stability. In addition, deheaded shrimp exhibited lower trypsin and cathepsin activities, reduced accumulation of TCA-soluble peptides and TVB-N, higher fluorescence intensity, and less severe degradation of myofibrillar proteins and muscle microstructure. These results collectively suggest that the cephalothorax accelerated post-mortem deterioration of shrimp muscle during cold storage.

The cephalothorax, particularly the hepatopancreas, contains high concentrations of endogenous proteases. After death, tissue disruption and lysosomal rupture promoted the release of trypsin and cathepsins into adjacent muscle, accelerating the degradation of myofibrillar proteins. This was evidenced by the significantly higher protease activities in the CK and IR groups, together with the rapid increase in TCA-soluble peptides and free amino acids during storage. Correspondingly, SDS-PAGE showed more pronounced weakening of actin and MLC bands and greater accumulation of low-molecular-weight peptides in headed shrimp. Similar structural deterioration was also observed at the microstructural level, where the CK and IR groups exhibited severe myofibrillar fragmentation, enlarged extracellular spaces, and disappearance of Z-lines. Relevant studies have shown that endogenous proteases released from the cephalothorax may initiate rapid post-mortem protein degradation, generating peptides and amino acids that provide favorable substrates for microbial growth [19,49]. Subsequently, spoilage bacteria such as *Pseudomonas* and *Shewanella* might further secrete extracellular proteases and metabolize amino acids into ammonia, biogenic amines, and other volatile spoilage compounds [19,49]. This process was reflected in the faster accumulation of TVB-N and bitter-related free amino acids, as well as the more severe sensory deterioration observed in the CK and IR groups.

Although removing the shrimp head removes most proteases and delays quality deterioration, endogenous proteases are still present in the muscle. In the later stages of cold storage, rapid microbial growth and metabolic activities lead to protein degradation. At the same time, the high content of polyunsaturated fatty acids in shrimp meat readily undergoes oxidation with oxygen during cold storage, producing hydroperoxides that subsequently decompose into small molecules like aldehydes, ketones, and acids, creating a rancid or off-flavor [10]. Furthermore, the muscle tissue of shrimp releases exudate under sealed static storage conditions, which is inevitable. During refrigeration, the accumulated exudate mainly consists of muscle water, soluble proteins, amino acids, and small molecular metabolites [19]. On one hand, the exudate creates a high-humidity microenvironment within the sealed bag, which accelerates the reproduction of spoilage microorganisms and promotes the activity of endogenous enzymes in the shrimp muscle, jointly accelerating the softening of the meat and the deterioration of flavor [65]. On the other hand, the loss of soluble nutrients and water leads to a decrease in the juiciness and sensory quality of the shrimp [19,66].

Wang et al. [12] demonstrated that the shrimp intestine significantly affected muscle quality deterioration during cold storage. However, only limited differences were observed between the CK and IR groups and between the HR and HIR groups in the present study. This discrepancy may be related to differences in pretreatment conditions. In Wang et al. [12] shrimp were peeled and sterilized with chlorine dioxide solution, which largely eliminated surface microorganisms and made intestinal microbiota the dominant contamination source. In contrast, the shrimp used in the present study were neither peeled nor sterilized before storage, resulting in a relatively high indigenous microbial load on the shrimp body. In addition, residual intestinal microorganisms may still have remained after intestine removal, thereby reducing the relative contribution of the intestine compared with the cephalothorax.

From an industrial perspective, cephalothorax removal represents a simple, economical, and chemical-free preservation strategy that effectively delayed protein degradation, melanosis, and sensory deterioration during refrigerated storage. Although the present study focused on *Litopenaeus vannamei*, hepatopancreas-mediated deterioration is common among shrimp species. Therefore, cephalothorax removal may also have preservation potential for other shrimp species, although its effectiveness may vary depending on species characteristics, body size, and initial microbial load.

## 5. Conclusions

To elucidate the effects of deheading and intestinal removal on protein degradation and quality changes in chilled *Litopenaeus vannamei*, shrimp were subjected to four treatments: control (CK, intact), heads removed (HR), intestines removed (IR), and both heads and intestines removed (HIR). The results showed that processing treatments significantly influenced endogenous protease activity and the extent of protein degradation in shrimp muscle during chilled storage. No significant differences were observed between the CK and IR groups or between the HR and HIR groups across the evaluated indices. In contrast, the HR and HIR treatments consistently exhibited superior preservation of muscle quality, extending the shelf life by 2 days, indicating that the hepatopancreatic tissue in the cephalothorax exerts a more pronounced effect on postmortem quality changes than the intestine. Removal of the cephalothorax effectively suppressed the increase in trypsin and cathepsin activities, including cathepsin B, B+L, and D, in the muscle. This was accompanied by a delayed accumulation of spoilage-related indicators, such as total volatile basic nitrogen, trichloroacetic acid-soluble peptides, and bitter amino acids including His, Lys, Leu, Val, and Tyr. In addition, deheading mitigated the disruption of myofibrillar microstructure and slowed the degradation of myofibrillar proteins. Overall, quality deterioration in shrimp is primarily driven by the hepatopancreatic tissue located in the cephalothorax. Its removal may limit the transfer of endogenous proteases into the muscle, thereby inhibiting protein degradation, delaying spoilage progression, and maintaining physicochemical stability and sensory quality during cold storage.

## Figures and Tables

**Table 1:** Quality descriptions and scores for sensory evaluation of *Litopenaeus vannamei*.

Quality Attributes	Description	Score
Appearance	Large areas of black spots or redness appear.	0–3
	Slightly red or black, with black spots on the head and tail.	4–6
	Bright luster, no black spots.	7–9
Texture	The muscle tissue is not tight, loose and has no elasticity.	0–3
	Not tight but not loose, the muscles become soft and have poor elasticity.	4–6
	Dense, complete, firm and elastic.	7–9
Odor	There is a distinct unpleasant smell, a putrid stench.	0–3
	The inherent smell of shrimp is quite distinct, with a slight off-flavor.	4–6
	The inherent smell of fresh shrimp, without any off-flavors.	7–9
Overall acceptability	The degree of corruption is serious.	0–3
	The freshness has deteriorated.	4–6
	Good freshness.	7–9

**Table 2:** Changes in color of *Litopenaeus vannamei* with different treatments during 4 °C.

	Group	Storage Time (d)						
		0	1	2	3	4	5	6
L*	CK	37.35 ± 0.48 ^Ad^	39.08 ± 0.44 ^Ab^	40.82 ± 0.64 ^Aa^	38.28 ± 0.58 ^Bc^	37.14 ± 0.69 ^Bd^	35.65 ± 0.76 ^BCe^	33.08 ± 0.73 ^Bf^
	HR	37.06 ± 0.69 ^Ac^	38.57 ± 0.33 ^Ab^	40.13 ± 0.63 ^Aa^	40.43 ± 1.00 ^Aa^	38.62 ± 0.77 ^Ab^	36.94 ± 0.57 ^Ac^	35.39 ± 0.59 ^Ad^
	IR	36.83 ± 0.88 ^Ac^	38.40 ± 0.75 ^Ab^	40.68 ± 0.42 ^Aa^	38.96 ± 0.78 ^Bb^	36.32 ± 0.69 ^Bc^	34.97 ± 0.30 ^Cd^	32.54 ± 0.41 ^Be^
	HIR	37.20 ± 0.43 ^Ac^	38.36 ± 0.43 ^Ab^	40.38 ± 1.02 ^Aa^	40.25 ± 0.75 ^Aa^	38.90 ± 0.67 ^Ab^	36.23 ± 0.62 ^ABd^	34.70 ± 0.53 ^Ae^
a*	CK	−1.17 ± 0.11 ^Af^	−1.10 ± 0.16 ^Af^	0.49 ± 0.15 ^Ae^	1.40 ± 0.20 ^Ad^	3.00 ± 0.30 ^Ac^	3.85 ± 0.20 ^Ab^	5.12 ± 0.23 ^Aa^
	HR	−1.18 ± 0.10 ^Af^	−1.15 ± 0.10 ^Af^	0.28 ± 0.14 ^Be^	0.76 ± 0.15 ^Bd^	1.72 ± 0.27 ^Bc^	2.41 ± 0.31 ^Bb^	3.38 ± 0.24 ^Ba^
	IR	−1.24 ± 0.07 ^Af^	−1.14 ± 0.12 ^Af^	0.45 ± 0.19 ^ABe^	1.54 ± 0.21 ^Ad^	2.89 ± 0.22 ^Ac^	4.23 ± 0.43 ^Ab^	5.35 ± 0.36 ^Aa^
	HIR	−1.16 ± 0.10 ^Af^	−1.13 ± 0.11 ^Af^	0.26 ± 0.13 ^Be^	0.88 ± 0.11 ^Bd^	1.49 ± 0.29 ^Bc^	2.67 ± 0.33 ^Bb^	3.66 ± 0.25 ^Ba^
b*	CK	1.36 ± 0.09 ^Af^	1.33 ± 0.24 ^Af^	2.08 ± 0.10 ^Ae^	2.95 ± 0.25 ^Ad^	4.19 ± 0.23 ^Ac^	5.96 ± 0.22 ^Ab^	8.79 ± 0.24 ^Ba^
	HR	1.32 ± 0.13 ^Af^	1.34 ± 0.16 ^Af^	1.84 ± 0.27 ^Be^	2.48 ± 0.28 ^Bd^	3.13 ± 0.32 ^Bc^	3.69 ± 0.28 ^Bb^	4.85 ± 0.22 ^Ca^
	IR	1.26 ± 0.13 ^Af^	1.52 ± 0.12 ^Af^	2.04 ± 0.15 ^ABe^	2.96 ± 0.23 ^Ad^	4.06 ± 0.30 ^Ac^	6.27 ± 0.55 ^Ab^	9.21 ± 0.31 ^Aa^
	HIR	1.36 ± 0.08 ^Af^	1.38 ± 0.08 ^Af^	1.93 ± 0.14 ^ABe^	2.36 ± 0.20 ^Bd^	3.05 ± 0.26 ^Bc^	4.06 ± 0.26 ^Bb^	5.14 ± 0.31 ^Ca^

(Note: Different capital letters indicate significant differences between groups (*p* < 0.05); while different lowercase letters indicate significant differences within groups (*p* < 0.05); CK, HR, IR, and HIR represent the pretreatment of shrimp as intact shrimp, shrimp with head removed, shrimp with intestine removed, and shrimp with head and intestine removed, respectively, followed by storage at 4 °C).

**Table 3:** Changes in free amino acid content of *Litopenaeus vannamei* with different treatments during 4 °C.

FAAs (mg/100 g)	Group								
	Fresh	D3-CK	D3-HR	D3-IR	D3-HIR	D6-CK	D6-HR	D6-IR	D6-HIR
**Asp**	13.83 ± 2.76 ^c^	7.69 ± 0.86 ^e^	2.61 ± 0.88 ^f^	9.93 ± 1.01 ^d^	3.84 ± 1.45 ^f^	20.51 ± 0.42 ^b^	9.58 ± 0.41 ^de^	26.24 ± 1.25 ^a^	13.10 ± 0.41 ^c^
**Ser**	18.79 ± 2.04 ^d^	32.44 ± 2.02 ^a^	23.29 ± 1.06 ^b^	31.53 ± 0.36 ^a^	22.72 ± 2.35 ^bc^	20.08 ± 2.16 ^cd^	13.77 ± 1.54 ^e^	20.56 ± 0.65 ^bcd^	14.21 ± 0.31 ^e^
**Glu**	147.32 ± 29.48 ^cd^	149.63 ± 17.28 ^cd^	127.08 ± 3.81 ^de^	151.01 ± 4.31 ^cd^	110.70 ± 11.97 ^e^	163.56 ± 3.29 ^bc^	178.46 ± 21.74 ^b^	225.78 ± 9.05 ^a^	174.51 ± 3.90 ^bc^
**Gly**	454.43 ± 15.41 ^a^	406.98 ± 16.31 ^c^	398.40 ± 9.13 ^c^	434.78 ± 2.65 ^b^	403.54 ± 7.6 ^c^	401.36 ± 0.56 ^c^	402.15 ± 14.15 ^c^	401.56 ± 4.50 ^c^	416.10 ± 1.65 ^c^
**Ala**	234.87 ± 17.29 ^d^	230.88 ± 15.56 ^d^	225.23 ± 6.41 ^d^	268.61 ± 4.11 ^c^	221.87 ± 14.17 ^d^	288.00 ± 6.11 ^bc^	298.89 ± 20.50 ^ab^	314.39 ± 6.96 ^a^	292.11 ± 3.87 ^b^
**Cys**	0.40 ± 0.02 ^a^	0.82 ± 0.74 ^a^	1.08 ± 0.85 ^a^	0.37 ± 0.03 ^a^	0.42 ± 0.04 ^a^	0.93 ± 1.07 ^a^	1.02 ± 0.70 ^a^	0.72 ± 0.01 ^a^	0.48 ± 0.02 ^a^
**Val**	30.54 ± 0.44 ^e^	40.78 ± 3.09 ^cd^	37.18 ± 0.8 ^d^	46.33 ± 2.13 ^b^	29.30 ± 1.69 ^e^	48.43 ± 1.34 ^b^	41.47 ± 4.78 ^c^	65.83 ± 2.12 ^a^	39.82 ± 0.41 ^cd^
**Met**	5.88 ± 0.53 ^f^	15.56 ± 0.89 ^d^	10.39 ± 0.3 ^e^	18.79 ± 0.31 ^c^	9.82 ± 1.00 ^e^	23.07 ± 0.27 ^b^	15.42 ± 1.36 ^d^	24.94 ± 0.59 ^a^	15.26 ± 0.22 ^d^
**Ile**	14.09 ± 0.44 ^f^	22.49 ± 2.09 ^d^	17.74 ± 0.84 ^e^	26.41 ± 0.87 ^c^	15.01 ± 1.14 ^f^	29.91 ± 1.79 ^b^	20.87 ± 2.39 ^d^	37.92 ± 1.17 ^a^	21.14 ± 0.12 ^d^
**Leu**	23.39 ± 0.45 ^f^	37.03 ± 3.52 ^d^	29.85 ± 1.19 ^e^	44.09 ± 1.39 ^c^	24.83 ± 2.04 ^f^	48.95 ± 2.77 ^b^	35.81 ± 4.59 ^d^	67.07 ± 2.34 ^a^	34.86 ± 0.29 ^d^
**Tyr**	21.33 ± 1.35 ^f^	29.58 ± 1.97 ^cd^	22.60 ± 0.91 ^f^	32.71 ± 1.32 ^bc^	19.72 ± 2.03 ^f^	32.99 ± 3.12 ^b^	26.09 ± 2.50 ^e^	43.69 ± 1.35 ^a^	27.17 ± 0.56 ^de^
**Phe**	14.67 ± 1.51 ^f^	27.21 ± 2.04 ^d^	20.44 ± 0.54 ^e^	35.79 ± 0.94 ^c^	18.37 ± 1.76 ^e^	41.99 ± 2.78 ^b^	24.54 ± 2.28 ^d^	50.01 ± 1.31 ^a^	25.41 ± 0.39 ^d^
**Lys**	58.73 ± 5.15 ^de^	72.01 ± 4.7 ^c^	61.02 ± 1.83 ^d^	74.33 ± 0.81 ^c^	51.80 ± 4.02 ^e^	84.57 ± 6.72 ^b^	66.20 ± 8.64 ^cd^	95.75 ± 1.58 ^a^	63.59 ± 1.17 ^d^
**His**	25.72 ± 1.94 ^d^	28.97 ± 2.25 ^cd^	27.04 ± 0.73 ^d^	32.64 ± 0.73 ^bc^	22.13 ± 1.19 ^f^	33.85 ± 3.53 ^b^	29.43 ± 3.16 ^cd^	40.03 ± 1.99 ^a^	29.05 ± 0.55 ^cd^
**Arg**	577.82 ± 27.39 ^a^	521.70 ± 37.27 ^b^	504.78 ± 22.59 ^bc^	539.59 ± 6.48 ^b^	469.86 ± 21.30 ^cd^	390.93 ± 23.02 ^e^	402.61 ± 24.83 ^e^	455.36 ± 3.71 ^d^	448.32 ± 4.99 ^d^
**Pro**	546.37 ± 85.09 ^a^	494.06 ± 39.22 ^ab^	415.52 ± 20.02 ^cde^	456.50 ± 21.89 ^bc^	355.95 ± 42.86 ^e^	379.39 ± 40.68 ^de^	409.58 ± 35.02 ^cde^	442.42 ± 12.98 ^bcd^	441.44 ± 2.08 ^bcd^

(Note: Different lowercase letters within the same row indicate significant differences at *p* < 0.05; CK, HR, IR, and HIR represent the pretreatment of shrimp as intact shrimp, shrimp with head removed, shrimp with intestine removed, and shrimp with head and intestine removed, respectively, followed by storage at 4 °C).

## Data Availability

The original contributions presented in this study are included in the article. Further inquiries can be directed to the corresponding authors.

## Abbreviations

The following abbreviations are used in this manuscript:

SDS	Sodium Dodecyl Sulfate
SDS-PAGE	Sodium dodecyl sulfate polyacrylamide gel electrophoresis
EDTA	Ethylene Diamine Tetraacetic Acid
Tris	Tris(hydroxymethyl)aminomethane
Bis-Tris	Bis-(2-Hydroxyethyl)amino-tris (Hydroxymethyl)methane
DTT	Dithiothreitol
AMC	7-Amino-4-methylcoumarin
DMSO	Dimethyl sulfoxide
BAPNA	N-Benzoyl-DL-arginine-4-nitroanilide hydrochloride
rpm	revolutions per minute
